# Role of hyperhomocysteine, thyroid dysfunction and their interaction in ischemic stroke patients with non-valvular atrial fibrillation

**DOI:** 10.1038/s41598-020-69449-2

**Published:** 2020-07-24

**Authors:** Lili Wang, Yun Zhang

**Affiliations:** Department of Neurology, Beijing Shijitan Hospital, Capital Medical University, Beijing, 100038 China

**Keywords:** Diseases, Neurology

## Abstract

The role of hyperhomocysteinemia (HHcy) and thyroid dysfunction in ischemic stroke with non-valvular atrial fibrillation (AF) remains controversial. Till now, the relationship between HHcy and thyroid dysfunction in ischemic stroke with non-valvular AF has not been reported. The aim of this study was to investigate the role and relationship of HHcy and thyroid dysfunction in ischemic stroke patients with non-valvular AF. Seven hundred and seventeen patients with acute ischemic stroke within the first 7 days of symptom onset were consecutively included in this study. Eligible patients were divided into AF group and without AF group. Variables including age, sex, smoke, drink, history of stroke were collected. Levels of homocysteine (Hcy), thyroid-stimulating hormone were evaluated at presentation. Multivariable logistic regression and spearman bivariate correlate analysis were used to evaluate the relationship of HHcy and thyroid dysfunction in ischemic stroke patients with AF. There were 122 patients with AF and 595 patients without AF. Two hundred and eighty-eight (40.2%) patients had HHcy and Three hundred and sixteen (44.1%) patients had thyroid dysfunction. There was significant difference of serum Hcy (*P* = 0.014) and thyroxine levels (*P* = 0.002) between patients with and without AF. Furthermore, the difference of serum Hcy (*P* = 0.007) and thyroxine levels (*P* = 0.004) between patients with and without AF was also significant in female subgroups. We did not find association between HHcy and thyroid dysfunction in AF group (*P* = 0.463). In conclusion, both HHcy and thyroid dysfunction were associated with AF in patients with acute ischemic stroke and in female subgroup patients. However, thyroid dysfunction had no relationship with HHcy in ischemic stroke patients with AF.

## Introduction

Homocysteine (Hcy) is a sulfur-containing amino acid produced by the demethylation of amino acid methionine^1^. Much studies have shown that elevated serum Hcy which is called Hyperhomocysteinemia (HHcy), arising from impaired methionine metabolism, is a risk factor for ischemic stroke^2-4^. Cardiogenic cerebral embolism caused by non-valvular atrial fibrillation (AF) has a worse prognosis and higher disability rate than other types of cerebral infarction. Risk factors related to cerebral embolism caused by non-valvular AF need to be identified. Recently, evidence has demonstrated the relationships between HHcy and AF^5^. Some researchers think HHcy may be one of the explanations for AF related thromboembolic complications^6,7^. Other researchers disagree with that HHcy may be a risk factor for stroke and thromboembolism in patients with non-valvular AF^8^. But to date, the relationship of HHcy with non-valvular AF in ischemic stroke remains controversial.

Thyroid hormones have a complex relationship with ischemic stroke. Data regarding the association between thyroid function and outcomes after ischemic stroke are contradictory^9,10^. Hyperthyroidism is associated with AF and cardio embolic stroke^11^. Subclinical hyperthyroidism is a risk factor for poor outcome 3 months after ischemic stroke^12^.

Recent works have revealed that overt hypothyroidism modulates Hcy levels by modulating gene expression and by influencing several enzymes involved in Hcy metabolism^11^. However, subclinical hypothyroidism was not associated with HHcy. Furthermore, study found HHcy was not associated with ischemic stroke patients with hypothyroidism^1^.

The role of HHcy and thyroid dysfunction in ischemic stroke with non-valvular AF was not fully elucidated. Till now, no study has demonstrated the relationship of thyroid dysfunction and HHcy in ischemic stroke with non-valvular AF. Whether HHcy and thyroid dysfunction can serve as the therapy targets in ischemic stroke with AF needs further investigation. The present study aimed to investigate how these abnormalities and their interaction may be involved in this ischemic stroke group.

## Results

### Patient characteristics

The clinical characteristics of the AF and NAF group were shown in Table 1. We collected 717 patients with acute ischemic stroke (within 7 days after onset) in this study. There were 122 patients with AF and 595 patients without AF. AF group of patients comprised 51 (41.8%) women and 71 (58.2%) men with a mean age of 76.6 years. NAF group included 187 (31.4%) women and 408 men (68.6%) with a mean age of 65.6 years.

**Table 1:** The relationship between serum homocysteine and ischemic stroke with non-valvular atrial fibrillation.

	AF (n = 122)	NAF (n = 595)	χ^2^	P1
Sex (man)	71 (58.2%)	408 (68.6%)	4.914	0.027
Age (>60 year)	109 (89.3%)	400 (67.2%)	24.048	0.000
Smoke	26 (21.3%)	205 (34.5%)	8.007	0.005
Drink	23 (18.9%)	133 (22.4%)	0.729	0.404
Abnormal thyroid function	74 (60.7%)	242 (40.7%)	16.402	0.000
history of stroke	46 (37.7%)	165 (27.7%)	4.849	0.030
HHcy	61 (50.0%)	227 (38.2%)	5.914	0.019

Logistic regression analysis for the relationship between serum homocysteine and ischemic stroke with non-valvular atrial fibrillation				
	P2	OR	95%CI	
Age	0.000	3.086	1.668–5.710	
Abnormal thyroid function	0.002	1.910	1.265–2.883	
HHcy	0.014	1.673	1.111–2.520	
Smoke	0.021	0.564	0.347–0.918	
History of stroke	0.090	1.443	0.944–2.204	
Sex	0.654	1.110	0.702–1.755	

*HHcy* hyperhomocysteinemia, *AF* atrial fibrillation, *NAF* non-atrial fibrillation

### Role of Hcy in ischemic stroke with non-valvular AF

The difference of serum Hcy level in AF and NAF groups is shown in Table 1. Patients in AF group were with higher proportion of female (*P* = 0.027), old age (*P* = 0.000), HHcy (*P* = 0.019), history of stroke (*P* = 0.030) and abnormal thyroid function (*P* = 0.000). Patients in NAF group were likely to smoke more cigarettes (*P* = 0.005). Logistic regression analysis showed abnormal thyroid function (*P* = 0.002, odds ratio, 1.910, 95% confidence interval, 1.265–2.883), smoke (*P* = 0.021, odds ratio, 0.564, 95% confidence interval, 0.347–0.918), HHcy (*P* = 0.014, odds ratio, 1.673, 95% confidence interval, 1.111–2.520) and age (*P* = 0.000 odds ratio, 3.086, 95% confidence interval, 1.668–5.710) had significant difference between AF and NAF groups. But history of stroke (*P* = 0.090) and sex (*P* = 0.654) did not show significant difference between the two groups.

### Role of serum Hcy in ischemic stroke with non-valvular AF in different sex groups

In female subgroup, patients with HHcy were significantly more common in AF group than in NAF group (47.1% versus 23.5%; *P* = 0.002). Logistic regression analysis showed the difference was still remarkable (*P* = 0.007; odds ratio, 2.472; 95% confidence interval, 1.275–4.794). However, in male subgroup, HHcy between AF and NAF group had no significant difference (*P* = 0.302). Abnormal thyroid function was more common in AF group than in NAF group in female patients (*P* = 0.004; odds ratio, 3.057; 95% confidence interval, 1.427–6.548). Baseline characteristics related to AF in different sex subgroups were show in Table2.

**Table 2:** The relationship between serum homocysteine and cerebral infarction patients with non-valvular atrial fibrillation in different sex groups.

	Male				Female			
	AF	NAF	χ^2^	P1	AF	NAF	χ^2^	P1
Age (> 60 year)	62 (87.3%)	249 (61.0%)	18.362	0.000	47 (92.2%)	151 (80.7%)	3.730	0.058
Smoke	23 (32.4%)	193 (47.3%)	5.430	0.020	3 (5.9%)	12 (6.4%)	0.019	1.000
Drink	19 (26.8%)	130 (31.9%)	0.735	0.409	4 (7.8%)	3 (1.6%)	5.464	0.040
HHcy	37 (52.1%)	183 (44.9%)	1.283	0.302	24 (47.1%)	44 (23.5%)	10.871	0.002
Abnormal thyroid function	33 (46.5%)	141 (34.6%)	3.715	0.061	41 (80.4%)	101 (54.0%)	11.588	0.001
history of stroke	28 (39.4%)	121 (29.7%)	2.699	0.126	18 (35.3%)	44 (23.5%)	2.879	0. 106

Logistic regression analysis for the relationship between serum homocysteine and cerebral infarction patients with non-valvular atrial fibrillation in different sex groups								
		P2	OR	95%CI				
Female	Abnormal thyroid function	0.004	3.057	1.427–6.548				
	HHcy	0.007	2.472	1.275–4.794				
	Drink	0.114	3.558	0.736–17.193				
Male	Age	0.000	4.173	2.012–8.659				
	Smoke	0.061	0.595	0.345–1.024				

*HHcy* hyperhomocysteinemia, *AF* atrial fibrillation, *NAF* non-atrial fibrillation

### Relationship of HHcy and thyroid dysfunction in ischemic stroke patients with AF

Table 3 showed the relationship of HHcy and thyroid dysfunction in the ischemic stroke patients with and without AF. There was no relationship between HHcy and thyroid dysfunction in AF group (*P* = 0.578) and NAF group (*P* = 0.303). In NAF group, HHcy was more common in female patients than in male patients (*P* = 0.000; odds ratio, 0.430; 95% confidence interval, 0.283–0.654). Spearman bivariate correlate analysis was further showed no relationship between HHcy and thyroid dysfunction (correlation coefficients = 0.067, *P* = 0.463) (Table 4).

**Table 3:** Relationship of hyperhomocysteinemia and thyroid dysfunction in ischemic stroke patients with atrial fibrillation.

	AF				NAF			
	NHHcy	HHcy	χ^2^	P1	NHHcy	HHcy	χ^2^	P1
Age (> 60 year)	52 (85.2%)	57 (93.4%)	2.152	0.240	244 (66.3%)	156 (68.7%)	0.373	0.590
Smoke	10 (16.4%)	16 (26.2%)	1.760	0.269	107 (29.1%)	98 (43.2%)	12.352	0.001
Drink	10 (16.4%)	13 (21.3%)	0.482	0.644	74 (20.1%)	59 (26.0%)	2.799	0.105
Sex (man)	27 (44.3%)	24 (39.3%)	0.303	0.714	143 (38.9%)	44 (19.4%)	24.709	0.000
Abnormal thyroid function	35 (57.4%)	39 (63.9%)	0.550	0.578	156 (42.4%)	86 (37.9%)	1.181	0.303
history of stroke	23 (37.7%)	23 (37.7%)	0.000	1.000	98 (26.6%)	67 (29.5%)	0.583	0. 452

Logistic regression analysis for the relationship between hyperhomocysteinemia and thyroid dysfunction in ischemic stroke patients with atrial fibrillation								
		P2	OR	95%CI				
NAF	Sex	0.000	0.430	0.283–0.654				
	Smoke	0.099	1.371	0.942–1.995				

*HHcy* hyperhomocysteinemia, *NHHcy* normal homocysteine, *AF* atrial fibrillation; *NAF* non-atrial fibrillation

**Table 4:** Relationship between HHcy and thyroid dysfunction in ischemic stroke patients with AF (Spearman bivariate correlate analysis).

	HHcy	
	Correlation coefficients	P
Abnormal thyroid function	0.067	0.463

*AF* atrial fibrillation, *HHcy* hyperhomocysteinemia

## Discussion

In this study, we found there was significant difference of serum Hcy, thyroxine levels, age and smoke between patients with and without AF. Furthermore, the difference of serum Hcy and thyroxine levels between patients with and without AF was also significant in female subgroups. In male subgroup, age difference was significant between patients with and without AF. But we found thyroid dysfunction had no relationship with HHcy in ischemic stroke patients with AF in this study.

Hcy is a risk factor for atherosclerosis and it also increased the rate of stroke in older patients with AF^7^. HHcy predicted severe neurological impairment and stroke recurrence in acute ischemic stroke subtype^13^. Plasma Hcy levels are influenced by age, gender and several other factors^14^. Till now, no report revealed the relationship between HHcy and AF in female patients with ischemic stroke. In this present study, we found that HHcy was associated with non-valvular AF in ischemic stroke patients, HHcy was also related to non-valvular AF in female stroke patients. Underlying mechanism including cell death signaling, immune response may contribute to the sex differences in ischemic stroke which need further identification^15^.

Hyperthyroidism is an important cause of AF and is associated with cardio embolic stroke^11,16,17^. Other studies suggested that thyroid hormones may be associated with sex, age and other factors to effect stroke outcomes^9^. In our study, thyroid dysfunction showed significant relationship with AF in patients with ischemic stroke, which was consistent with previous studies. In future, role of T3, T4, TSH in AF and cardio embolic stroke should be further studied respectively.

In this study, we found patients with age older than 60 years were more common in AF group than in non-atrial fibrillation group. We also found woman was more common in AF group than in non- AF group. This result was consistent with previous studies showing female ischemic stroke patients with more AF^18^. But in multivariable logistic regression analysis, the sex difference did not show significance. Due to the incidence rates of thromboembolism were higher in Chinese female patients with AF compared with male patients^19^, further study should focus on the mechanism of cardio-embolic ischemic stroke in women.

There is enough evidence that hypothyroidism is associated with HHcy^11^. HHcy is a risk factor for ischemic stroke and hypothyroidism is associated with ischemic stroke. Hypothyroidism may cause HHcy. But HHcy was not found to be associated with ischemic stroke patients with hypothyroidism^1^. Till now, no study has demonstrated the relationship of thyroid dysfunction and HHcy in ischemic stroke with non-valvular AF. The present study investigated the relationship and found that thyroid dysfunction was not associated with HHcy in AF group. Further study should focus on the relationship of T3, T4 and TSH respectively with HHcy in ischemic stroke with AF.

Our study has some limitations. First, our study was done in one hospital and involved a relatively small group of patients. Second, we did not investigate the association between HHcy and hypothyroidism/hypothyroidism respectively in ischemic stroke with AF.

In conclusion, our results showed HHcy and thyroid dysfunction were both associated with AF in patients with acute ischemic stroke and in female subgroup patients. But thyroid dysfunction was not associated with HHcy in ischemic stroke patients with non-valvular AF. To the best of our knowledge, this is the first study that investigated the relationship between HHcy and thyroid dysfunction in ischemic stroke patients with non-valvular AF. Our data suggest that HHcy and thyroid dysfunction can serve as the risk factors therapy targets in ischemic stroke with AF. Studied should be conducted in larger patient group and at subgroup levels to further elucidate the relationship between thyroid dysfunction and HHcy.

## Methods

### Patients

Data of 717 eligible patients with acute ischemic stroke (within 7 days after onset) who were admitted to our hospital from July 2018 to December 2019 were prospectively collected in this study. Stroke was confirmed by magnetic resonance imaging (MRI) of the brain within 1 week of onset of symptoms. Patients with hemorrhagic stroke, venous infarcts, arterial dissection, Moyamoya disease or vasculitis were excluded. We also ruled out patients with impaired renal function. There were 122 patients with AF and 595 patients without AF. The study was approved by the ethics committee in our hospital. All patients provided written informed consent before enrolment. All methods were carried out in accordance with relevant guidelines and regulations.

### Baseline characteristics

Baseline information was collected at admission including age, gender, AF, history of stroke, HHcy and thyroid function. History of stroke was defined as the experience of ischemic stroke. Smoke was defined as smoking continuously ≥ 1 cigarette a day for at least 1 year. Drink was defined as drinking continuously ≥ 30 g/week for more than 1 year. AF was confirmed by Electrocardiograph (ECG). Serum Hcy levels were determined by high performance liquid chromatography^20^. HHcy was defined as total serum Hcy  > 16 mmol/L at the time of admission. Thyroid diseases including Graves’ disease, Hashimoto thyroiditis and other antibody positive thyroid diseases were diagnosed on the basis of clinical features, thyroid ultrasonography, serum thyroxin level and related autoantibodies. It was identified as an abnormal thyroid function if one of the following serum thyroxin or related autoantibodies including total thyroid hormone T3 (TT3), total thyroid hormone T4 (TT4), free thyroid hormone T3 (FT3), free thyroid hormone T4 (FT4), thyroid stimulating hormone (TSH), thyroglobulin antibody (ATG), thyroid peroxidase antibody (ATA) is beyond the normal level at the time of admission.

### Statistical analysis

SPSS software version22 (IBM, New York) was used for statistical analysis. χ^2^ test was used to compare categorical variables. Variables that were identified as significant in the univariate analysis (P < 0.05) were used in multivariable logistic regression analysis to examine their independent roles. Spearman bivariate correlate analysis was further used to test the relationship between HHcy and thyroid dysfunction. All tests were 2-sided, P < 0.05 was considered statistically significant.

### Ethics approval and consent to participate

The study protocols were approved by the ethics committees of Beijing Shijitan Hospital, Capital Medical University. All patients participating in the study had written informed consent.
